# The Prevalence, Pathophysiological Role and Determinants of Mitral Annular Disjunction Among Patients with Mitral Valve Prolapse: A Systematic Review

**DOI:** 10.3390/jcm14051423

**Published:** 2025-02-20

**Authors:** Andrea Sonaglioni, Gian Luigi Nicolosi, Giovanna Elsa Ute Muti-Schünemann, Michele Lombardo, Paola Muti

**Affiliations:** 1 Division of Cardiology, IRCCS MultiMedica, 20123 Milan, Italy; michele.lombardo@multimedica.it; 2 Division of Cardiology, Policlinico San Giorgio, 33170 Pordenone, Italy; gianluigi.nicolosi@gmail.com; 3 Department of Emergency, Fondazione IRCSS Ca’ Granda, Ospedale Maggiore Policlinico, 20122 Milan, Italy; giovanna.muti@unimi.it; 4 Department of Biomedical, Surgical and Dental Sciences, University of Milan, 20122 Milan, Italy; pmuti26@gmail.com; 5 IRCCS MultiMedica, 20138 Milan, Italy

**Keywords:** mitral annular disjunction, mitral valve prolapse, mitral regurgitation, ventricular arrhythmias, outcome

## Abstract

**Background:** Over the last two decades, a number of imaging studies have evaluated the characteristics and clinical implications of mitral annular disjunction (MAD) among patients with mitral valve prolapse (MVP). The present systematic review has been primarily designed to summarize the main findings of these studies and to examine the overall impact of MAD in MVP patients. **Methods:** All imaging studies assessing the prevalence, pathophysiological role and determinants of MAD in MVP individuals, selected from the PubMed and EMBASE databases, were included. There was no limitation in terms of time period. The risk of bias was assessed by using the National Institutes of Health (NIH) Quality Assessment Tool for Observational Cohort and Cross-Sectional Studies. **Results:** The full texts of 23 studies on 7718 MVP individuals were analyzed. The overall pooled prevalence of MAD in MVP individuals was 40% (range 5.4–90%). When considering the different imaging modalities for assessing MAD, the average MAD prevalence was 20% for cardiac computed tomography studies, 31.3% for transthoracic echocardiography (TTE) studies, 44.7% for transesophageal echocardiography studies and 47% for cardiac magnetic resonance studies. MAD presence was more commonly associated with female sex, young age, narrow antero-posterior thoracic diameter, symptoms of palpitations and syncope, T-wave inversion in inferolateral leads and frequent and/or complex ventricular arrhythmias (VAs) on electrocardiogram, myxomatous leaflets, bileaflet prolapse, larger mitral valve annulus and non-severe mitral regurgitation on TTE. A total of 12 studies (52.2%) provided follow-up data. Over a median follow-up time of 3.9 yrs (range 1–10.3 yrs), MVP individuals with MAD showed increased risk of clinical arrhythmic events, no difference in survival rate and good surgical outcomes. **Conclusions:** MAD was present in more than one-third of MVP patients, with a wide range of variability depending on the specific imaging method used for assessing MAD presence and on a nonunivocal MAD definition, with a possible overestimation due to Pseudo-MAD rather than True-MAD measurement. A multimodality imaging approach comprehensive of noninvasive chest shape assessment might improve MAD detection among MVP individuals. It appears that careful serial monitoring for VAs should be mandatory for MAD patients.

## 1. Introduction

Mitral valve prolapse (MVP) is the most frequent cause of primary mitral regurgitation (MR), affecting about 2–3% of the general population [1,2,3]. MVP is diagnosed as the systolic displacement of one or both leaflets by at least 2 mm above the annular plane into the left atrium, in the parasternal long-axis view [2,4].

Even if MVP is generally considered a benign condition, a subset of individuals may be affected by the arrhythmic MVP that carries an increased risk of complex ventricular arrhythmias (VAs) and sudden cardiac death [5]. The mechanisms for arrhythmias in patients with MVP are still not completely understood. According to the most recent literature, severe alterations in mitral valve structure, including bileaflet prolapse, a myxomatous mitral valve with thickened leaflets and mitral annular disjunction (MAD), as well as the presence and extent of myocardial fibrosis (MF) at late gadolinium enhancement (LGE) cardiac magnetic resonance (CMR), have been associated with increased arrhythmic risk in MVP individuals [6,7,8].

MAD is a structural abnormality, defined as the atrial displacement of the hinge point of the mitral annulus from the underlying ventricular myocardium [9]. It has been closely linked to MVP. However, some reports indicate that MAD also may appear without concomitant MVP [10]; an MAD distance of 3 mm or less may be a benign finding in patients without mitral valve (MV) disease [11].

From a pathophysiological point of view, MAD may contribute to paradoxical systolic expansion of the mitral valve annulus (MVA) [12], as well as to myxomatous degeneration of the leaflets, with consequent MR progression [13]. Due to the abnormal tugging on the submitral apparatus resulting in MF, usually located in the inferolateral basal wall of the left ventricle, MAD may trigger complex VAs [6,7,14]. The wider the magnitude of the disjunction, the higher the incidence of VAs [15].

MAD may be assessed by transthoracic echocardiography (TTE), transesophageal echocardiography (TEE), cardiac computed tomography (CCT) or CMR. Due to its excellent spatial resolution, CCT may theoretically allow the most accurate assessment of the circumferential extent and the severity of MAD [11]. However, to date, there are no studies that have compared MAD diagnosis based on CCT to that based on other imaging modalities. CMR is able not only to precisely quantify the MAD extent, but also to provide an accurate assessment of the pattern and distribution of myocardial fibrosis at LGE imaging [16]. Compared to CCT and CMR, TTE suffers from lower spatial orthogonal resolution and limited imaging views [17].

Over the last two decades, a number of imaging studies have evaluated the prevalence, pathophysiological role and determinants of MAD among MVP patients. The present systematic review aimed at summarizing the main findings of these studies and examining the overall impact of MAD in patients with MVP.

## 2. Materials and Methods

This systematic review was performed according to the PRISMA guidelines [18], and is registered in the PROSPERO database (CRD42024570888).

### 2.1. Search Strategy

A comprehensive search of all imaging studies assessing MAD in MVP patients, regardless of time frame, was carried out by two independent reviewers (A.S. and M.L.), by using the Medline and EMBASE databases. The search strategy included the following terms: “mitral annular disjunction” OR “MAD” AND “mitral valve prolapse” OR “MVP” AND “prevalence” AND “outcome” AND “cardiac function” AND “left ventricular mechanics” AND “ventricular arrhythmias” AND “mitral regurgitation” AND “transthoracic echocardiography” AND “transesophageal echocardiography” AND “cardiac computed tomography” AND “cardiac magnetic resonance” OR “CMR”. The search was limited to full-text articles published in English.

### 2.2. Eligibility Criteria

All imaging studies evaluating the prevalence, pathophysiological role and determinants of MAD among MVP patients were included. Conversely, imaging studies conducted on patients without MVP, imaging studies conducted on MVP patients that did not evaluate MAD and its determinants, non-clinical articles, animal studies, duplicate articles, case reports, reviews, editorials, research letters without data and abstracts were excluded.

### 2.3. Study Selection and Data Extraction

Two reviewers (A.S. and M.L.) screened the databases according to the inclusion criteria and performed data extraction independently. Information concerning the following were independently collected by the two reviewers: (1) demographics (age and sex); (2) anthropometrics, such as body surface area (BSA), body mass index (BMI) and chest wall conformation, as noninvasively assessed by modified Haller index (MHI) [19]; (3) prevalence of the most relevant cardiovascular risk factors (smoking, hypertension, dyslipidemia and type 2 diabetes mellitus); (4) history of coronary artery disease (CAD); (5) symptoms, such as dyspnea, palpitations, fatigue, chest pain and/or syncope; (6) electrocardiography (ECG) and/or 24 h Holter monitoring findings, such as the heart rate, the eventual presence of T-wave inversion in inferolateral leads and atrial and/or ventricular arrhythmias, including non-sustained ventricular tachycardia (NSVT), defined as three or more consecutive beats arising below the atrioventricular node, with an RR interval of <600 ms (>100 beats/min) and lasting <30 s [20]; (7) MVA diameters, MAD extent and MR degree; (8) main indices of cardiac morphology and function, assessed by imaging studies according to international guidelines [21,22]; (9) current medical treatment; and (10) follow-up data, if any. A third author (G.L.N.) checked the extracted data for accuracy and resolved possible discrepancies between reviewers. Given the non-normal distribution of all continuous data, the latter were expressed as the median and interquartile range, whereas categorical variables were presented as a number (percentage).

### 2.4. Risk of Bias Assessment

The articles included in our analysis were assessed for risk of bias (RoB) using the National Institutes of Health (NIH) Quality Assessment Tool for Observational Cohort and Cross-Sectional Studies [23]. The quality rating was independently estimated by two authors (A.S. and G.L.N.). Disagreement was resolved by consensus.

**Figure 1:**
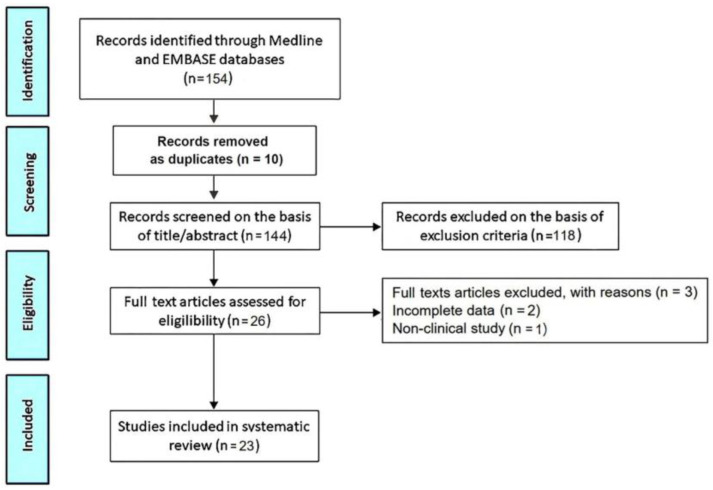
Flow diagram used for identifying included studies.

The PRISMA flow diagram used for identifying the included studies is depicted in Figure 1.

## 3. Results

The initial search yielded a total of 154 studies. Of these, 10 (6.5%) were removed as duplicates. After screening the titles and abstracts, a further 118 studies (76.6%) were removed on the basis of the exclusion criteria. The evaluation of the full texts of the remaining 26 studies (16.9%) resulted in a further three exclusions (1.9%). A total of 23 studies (14.9%) [7,15,16,24,25,26,27,28,29,30,31,32,33,34,35,36,37,38,39,40,41,42,43] were thus included in this systematic review, totaling 7718 MVP individuals.

### 3.1. Main Findings of Included Studies

A summary of the included studies’ characteristics and main findings is reported in Table 1.

The included studies were published between 2005 and 2025. Seven studies each were performed in the USA and Italy, two in France and one each in Canada, Portugal, the Principality of Monaco, the United Kingdom, Australia, Turkey, Belgium and the Netherlands, whereas the study of Figliozzi, S. et al. [7] was a multicenter European study. A total of 19 out of 23 studies (82.6% of total) were single-center studies. The great majority of studies (87% of total) had a retrospective design, whereas only 13% of the total had a prospective design. The mean age of MVP patients among the included studies was 55 yrs (range 12.8–82 yrs). Females accounted for 46.7% (range 18.3–75%) of MVP individuals examined by the included studies. All the included studies defined MVP as the systolic displacement of one or both leaflets by at least 2 mm above the MVA plane into the left atrium, assessed from the parasternal long-axis view during conventional TTE examination [2,4]. MAD was defined as the detachment of the left atrial (LA) wall–MVA junction from the LV myocardium, occurring during systole in the parasternal long-axis view [10], by 21 studies (91.3% of total). On the other hand, the most recent multicentric studies [42,43] distinguished between True-MAD (atrial displacement of the posterior leaflet in diastole and systole) and Pseudo-MAD (apparent displacement in systole only), and found a significantly lower prevalence of True-MAD vs. Pseudo-MAD in MVP individuals. MAD presence and extent was assessed by using only TTE in eleven studies (47.8% of total), only TEE in two studies (8.7% of total), only CMR in seven studies (30.4% of total) and only CCT in one study (4.3% of total), whereas two studies [16,43] assessed the agreement among echocardiographic techniques and CMR for MAD identification and measurement. Seven studies (30.4% of total) evaluated the MAD prevalence among MVP individuals with moderate-to-severe and/or severe MR who underwent MV surgery and had a pre-surgical evaluation by TTE, TEE, CMR or CCT, whereas the remaining sixteen studies (69.6% of total) were focused on MVP patients with mild and/or mild-to-moderate MR. Among the echocardiographic studies included, five (21.7% of total) performed a TTE examination implemented with speckle tracking echocardiography (STE) for evaluating left ventricular (LV)-global longitudinal strain (GLS) and regional longitudinal strain at the basal, mid- and apical level.

Table 2 lists all the relevant clinical characteristics of MVP patients and principal conventional and functional cardiac indices assessed by the different imaging studies in the same individuals.

### 3.2. Clinical Characteristics of MVP Patients

On average, the MVP patients examined by the included studies were middle-aged individuals, with a slightly increased prevalence of males (53.3% of total), with a low-to-moderate prevalence of the most common cardiovascular risk factors and a reduced burden of cardiovascular disease. Indeed, a history of previous CAD and previous congestive heart failure (CHF) could be detected in only 10.4% and 15.9% of MVP patients, respectively. The most commonly reported symptoms were palpitations (35.9%), followed by dyspnea (22.4%), fatigue (16.4%) and chest pain (16%). Concerning anthropometrics, MVP individuals with normal weight were found, as assessed by BSA and BMI; in addition, our study group observed that MVP individuals with MAD had a significantly shorter antero-posterior (A-P) thoracic diameter and significantly greater MHI than those without MAD [31].

### 3.3. ECG and/or 24-Hour Holter Monitoring Findings

On resting ECG, T-wave inversion in inferolateral leads was described in approximately one-third of MVP individuals. On 24 h Holter monitoring, ventricular and atrial arrhythmias were detected in 33.4% and 20% of the MVP patients included, respectively. Among VAs, NSVT was reported in 11.8% of MVP patients (range 1.8–33%).

### 3.4. Conventional Indices of Cardiac Size and Function

The LV wall thickness of MVP patients was normal, whereas LV internal dimensions were mildly increased with consequent eccentric LV remodeling. LA cavity size was moderately dilated. LV systolic function (assessed by left ventricular ejection fraction), LV filling pressures (expressed by the E/e’ ratio), RV size, RV systolic function (assessed by tricuspid annular plane systolic excursion) and pulmonary hemodynamics (measured by systolic pulmonary artery pressure) were normal.

### 3.5. Mitral Valve Parameters

Approximately two-thirds of MVP individuals (60.8% of total) were diagnosed with myxomatous MV morphology, and more than half of the total (55%) were affected by severe MR. The prevalence of bileaflet MVP (46%) and posterior MV leaflet prolapse (43.9%) was similar. MVA was mildly dilated [average value 35.9 mm (range 29.6–44.5 mm)]. The overall pooled prevalence of MAD in MVP individuals was 40% (range 5.4–90%). When considering the different imaging modalities for assessing MAD presence, CMR studies showed higher diagnostic sensitivity for detecting MAD. Notably, the average MAD prevalence was 20% for CCT studies, 31.3% (range 14.9–55%) for TTE studies, 44.7% (range 25.5–69.7%) for TEE studies and 47% (range 5.4–90%) for CMR studies. The pooled MAD extent was 7.1 mm (range 4.2–10 mm). The average percentage of females among MVP individuals with MAD was 60% (range 52–66%). A paradoxical systolic MVA expansion was described in MAD patients in three studies (13% of total) [15,27,33]. Finally, the Pickelhaube sign, defined as a systolic spike in the lateral mitral annulus on pulsed-wave (PW) tissue Doppler imaging (TDI) [44], was associated with MAD presence in 24.7% (range 6.5–37%) of MVP patients.

### 3.6. Myocardial Strain Parameters

STE examination, performed by a limited number of studies, showed that LV-GLS magnitude was mildly attenuated in MVP patients compared to the accepted reference values (more negative than −20%) [45]. Analysis of LV regional longitudinal strain in MVP patients revealed a significant impairment in LV-basal longitudinal strain (BLS), a mild reduction in LV-mid-longitudinal strain (MLS) and a normal and/or supranormal LV-apical longitudinal strain (ALS). The resultant physiological “base-to-apex gradient” (lowest-to-highest) in LV myocardial deformation was preserved. A concomitant impairment in LA strain in the reservoir phase was described in MVP individuals by two studies [37,39].

**Figure 2:**
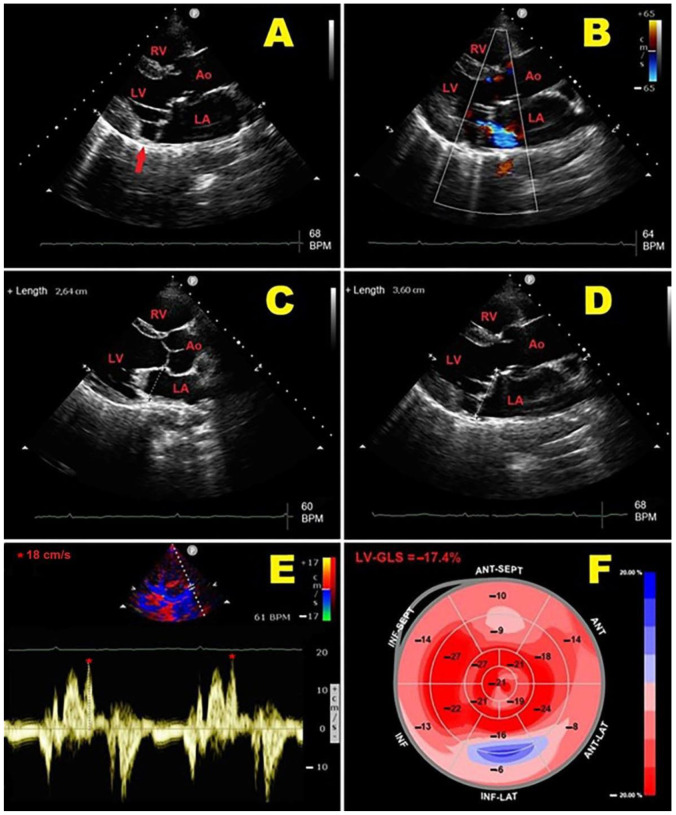
Representative example of main echocardiographic findings detectable in MVP individual with MAD. (**A**): Transthoracic parasternal long-axis view showing MAD (red arrow) and bileaflet MVP. (**B**): Transthoracic parasternal long-axis view showing bileaflet MVP with mild-to-moderate mitral regurgitation. (**C**): End-diastolic MVA diameter measurement from transthoracic parasternal long-axis view. (**D**): End-systolic MVA diameter measurement from transthoracic parasternal long-axis view, with evidence of paradoxical systolic MVA expansion. (**E**): PW-TDI of lateral mitral annulus, demonstrating high-velocity (>16 cm/s) mid-systolic spike in tissue Doppler velocity profile of mitral valve annulus (Pickelhaube sign). (**F**): LV-GLS bull’s eye plot assessed by 2D-STE analysis. In present case, mild impairment of LV-GLS (absolute value = −17.4%) was primarily related to LV-BLS attenuation, with peculiar involvement of LV-basal inferolateral wall. 2D, two-dimensional; Ao, aorta; BLS; basal longitudinal strain; GLS, global longitudinal strain; LA; left atrium; LV, left ventricle; MAD, mitral annular disjunction; MVA, mitral valve annulus; MVP, mitral valve prolapse; PW, pulsed-wave; RV, right ventricle; STE, speckle tracking echocardiography; TDI, tissue Doppler imaging. The symbol * indicates the mid-systolic peak velocity in tissue Doppler velocity profile of mitral valve annulus.

A representative example of the main echocardiographic findings detectable in an MVP individual with MAD is illustrated in Figure 2. 

**Figure 3:**
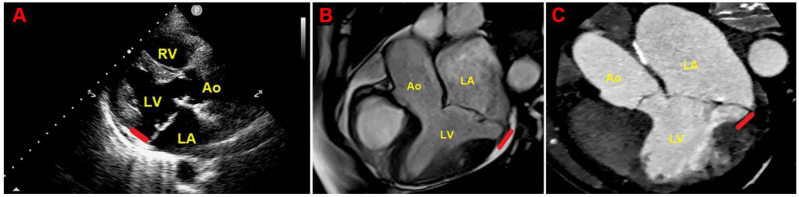
Example of MAD assessment at end-systole from echocardiographic parasternal long-axis view (**A**), from CMR-derived three-chamber view (**B**) and from multiplanar reconstructed three-chamber view on CCTA (**C**), in individual with bileaflet MVP. Ao, aorta; CCTA, cardiac computed tomography angiography; CMR, cardiac magnetic resonance; LA, left atrium; LV, left ventricle; MAD, mitral annular disjunction; MVP, mitral valve prolapse; RV, right ventricle. The red lines indicate the MAD distance.

Figure 3 depicts an example of MAD assessment at end-systole from TTE, CMR and CCT in an individual with bileaflet MVP.

### 3.7. CMR Findings

CMR studies demonstrated abnormal LGE of the LV-basal inferolateral wall and papillary muscles (PMs) in 35.9% of MVP individuals (range 6.1–68%).

Compared to MVP patients without MAD, those with MAD were more likely to have an abnormal basal inferolateral/papillary muscle LGE. MF, determined according to LGE presence and extent at CMR, was independently associated with increased risk of severe VAs and adverse outcomes over the follow-up period in MVP patients with MAD [7,26,33,36,38,41].

The authors ascribed the MF genesis to the traction exerted by MVP and MAD on the basal inferolateral/papillary muscles, generating an arrhythmogenic substrate and potentially triggering life-threatening VAs.

### 3.8. Current Medical Treatment

Approximately one-third of MVP patients were treated with beta blockers and/or ACE inhibitors, whereas antiarrhythmics were less frequently prescribed.

### 3.9. Follow-Up Data

A total of 12 studies (52.2%) provided follow-up data. The median follow-up time was 3.9 yrs (range 1–10.3 yrs). Overall, MVP patients with MAD were found to have increased risk of clinical arrhythmic events over the follow-up period. The wider the magnitude of the disjunction, the higher the incidence of complex and/or malignant VAs. Carmo, P. et al. [15] demonstrated that an MAD extent of >8.5 mm measured on TTE was able to predict the NSVT risk on 24 h Holter monitoring; moreover, Hussain, N. et al. [33] found that an MAD distance of ≥4 mm plus abnormal LGE assessed by CMR predicted an increased risk of malignant VAs. Palmisano et al. [42] reported the occurrence of sustained ventricular tachycardia and/or ventricular fibrillation and resuscitated sudden cardiac death in 11.5% of True-MAD patients with bileaflet MVP.

Three studies [28,33,38] reported that 2.3%, 3% and 12.5% of the whole cohort of MVP patients, respectively, underwent ventricular tachycardia ablation, particularly those with MAD.

The MAD extent was correlated with higher all-cause mortality risk over the follow-up period in two studies [35,40], whereas two authors [24,29] did not observe a statistically significant difference in survival between MVP patients with MAD and those without. Indeed, the studies of Eriksson, M.J. et al. [24] and Bennet, S. et al. [29], that evaluated MVP patients with moderate-to-severe and/or severe MR who underwent surgical intervention, demonstrated that MAD did not affect surgical outcomes and showed MAD disappearance in the great majority of MVP patients. The authors demonstrated that the current surgical techniques are able to correct the MAD abnormality in the vast majority of patients.

### 3.10. Risk of Bias

Regarding the RoB, the NIH quality rating was estimated as good for 14 studies and fair for the remaining 9 studies. The Cohen’s Kappa coefficient for the agreement between the reviewers in the RoB assessment indicated substantial agreement, κ = 0.81.

## 4. Discussion

### 4.1. MAD Prevalence Among MVP Individuals

The present systematic review, which analyzed the main findings of 21 imaging studies conducted on MVP individuals, demonstrated that the overall pooled prevalence of MAD in MVP individuals was 40%. Due to the significant heterogeneity between the included studies with regard to demographics, clinical characteristics of MVP individuals, degree of MVP and severity of MR associated with MVP, and the specific imaging method used for assessing MAD presence, the estimated MAD prevalence among MVP individuals showed a wide range of variability, ranging from 5.4% to 90%. An explanation for this finding is related to the possible overestimation of the real MAD prevalence due to Pseudo-MAD measurement by the great majority of included studies. Conversely, only the two most recent CMR studies were specifically focused on True-MAD identification, reporting a low prevalence of systolic and diastolic MAD among MVP individuals.

It is also important to consider that the MAD prevalence estimated by CMR studies turned out to be approximately 1.5-fold higher than that derived from TTE studies. Posterior MAD and MAD distances of <4 mm are frequently underdiagnosed by echocardiographic imaging. The lower detection rate of MAD by TTE has been ascribed to different mechanisms, such as an inadequate acoustic window, shadowing or reverberations caused by posterior MVA calcification and limited spatial lateral resolution [16]. Another limitation of TTE is the tendency to slightly overestimate the MAD distance compared to other imaging modalities. CMR is more accurate than echocardiography in detecting short-length MAD (typically <4 mm), due to its superior ability to visualize the posterior MVA and to assess its relationship with the LV wall and adjacent structures [46]. Moreover, CMR allows for the accurately identification of myocardial fibrosis in the LV-basal inferolateral wall and in the posterior papillary muscle by LGE [14,47]. However, the local availability of this technique, together with procedural costs, scanning and post-processing time duration, still limit the use of CMR in clinical routine. Based on the included studies’ findings, the MAD prevalence assessed by TEE studies turned out to be intermediate between TTE and CMR studies. It is noteworthy that TEE provides superior diagnostic accuracy for evaluating MV scallops, MVA diameters and MAD distance, compared to TTE [48]. Due to its semi-invasive nature, TEE is generally performed when TTE is inconclusive or technically difficult. Despite its limited sensitivity, TTE is still considered the modality of choice to assess MAD because of its widespread availability, low costs and excellent capacity to evaluate the haemodynamic consequences of MR [49]. Even if the present systematic review included only one CCT study [27], recent evidence indicates that MAD may be detected by CCT in more than 90% of individuals with structurally normal hearts, with bimodal distribution predominantly involving the P1 and P3 scallops of the posterior mitral leaflet [11].

### 4.2. Factors Associated with MAD Presence in MVP Individuals

MAD presence was associated with a number of demographics, anthropometrics, clinical characteristics of MVP individuals and typical MVP phenotypes, highlighted by each of the studies included in the present systematic review. Compared to MVP individuals without MAD, those with MAD were mostly females, were significantly younger, had a concave-shaped chest wall and/or narrow A-P thoracic diameter, were commonly symptomatic for palpitations, had a lower prevalence of the most relevant cardiovascular risk factors and had a lower comorbid disease burden [28]. MAD was more frequently detected in the LV inferolateral wall in comparison to other LV regions. Consistently with the literature data [11,12], circumferential and/or heterogeneous distribution of MAD was also described, particularly in MVP patients with severe valvulopathy [30]. However, recent evidence indicates that only extensive inferolateral disjunction is clinically relevant in MVP individuals, whereas disjunction elsewhere in the annulus should be considered a normal finding [50]. Female patients with MVP and MAD were less likely to be affected by severe MR [26,28,31]. Conversely, severe forms of MR were predominantly reported in males with advanced MVP and longer MAD extent [29,32,35]. MVP individuals with MAD with subclinical myocardial dysfunction were also found, defined by the reduced magnitude of myocardial strain parameters, in the presence of preserved LVEF (≥55%) [45]. Impairment in myocardial deformation indices was more enhanced at the level of mid- and basal segments, with peculiar involvement of the LV-basal inferolateral wall [31,37]. Consistently with STE results, CMR studies demonstrated abnormal LGE in the LV-basal inferolateral wall and PMs in MVP patients with MAD [26,33]; this finding was correlated with increased risk of malignant VAs [33,36,38].

### 4.3. Pathophysiology of MAD

Given that the most common site of origin of VAs in MVP individuals with MAD is the LV-basal inferolateral wall, it has been hypothesized that MAD and bileaflet prolapse lead to a systematic and repetitive stretch on the posterobasal myocardium or the PMs, which, over time, can induce the development of myocardial fibrosis [24,32,36,51]. The greater the amount of myocardial fibrosis, the higher the risk of malignant VAs in patients with MVP [14,41]. Especially pronounced MADs might play an arrhythmogenic role [52]. Areas of myocardial fibrosis may be detected by strain echocardiographic imaging [53,54,55] or assessed by LGE-CMR, which is actually considered as the gold-standard imaging technique to noninvasively identify and quantify myocardial fibrosis [56,57]. These areas of myocardial fibrosis are responsible for both the attenuation of myocardial strain parameters on STE and the abnormal LGE on CMR. The abnormal stretching of the LV-basal inferolateral wall and PMs induced by MAD is considered the causal factor for the repolarization abnormalities frequently detected in these individuals [58]. Hutchins et al. [10] hypothesized that the excessive mobility of the mitral valve apparatus secondary to disjunction of the mitral annulus fibrosus might cause repeated mechanical stress on mitral leaflets, leading to myxomatous valve degeneration.

Recent evidence derived from autoptic studies indicates that MAD may represent an anatomical variation, more frequently found at the P2 level (detected in approximately 20% of normal hearts), and more pronounced in individuals with prolapsing leaflets [59,60].

Our study group found a strong inverse correlation between MAD extent and the A-P thoracic diameter, indicating a possible relationship between MAD presence and chest wall conformation: the shorter the A-P thoracic diameter, the greater the MAD extent. Considering that the primordia of the mitral valve undergo differentiation to their final form at around the fifth to sixth week of gestation, the same period during which the vertebral column and the thoracic cage start their chondrification and ossification [61,62], we supposed that a defect in growth patterns at this stage of development might affect both the mitral valve and the bony thorax shape [63]. Therefore, it is likely that both MAD and the continuous mechanical stress perpetuated by the narrow A-P thoracic diameter might contribute to the myxomatous degeneration of MV. This “mechanical theory” would primarily explain the occurrence of ventricular and/or atrial arrhythmias and the reduced magnitude of basal longitudinal strain observed in MVP individuals with MAD, as secondary to extrinsic thoracic compression on cardiac chambers in the absence of any intrinsic myocardial dysfunction. As suggested by previous authors [64,65], it is also possible that a concave-shaped chest wall and/or pectus exacavatum, MVP and MAD may be altogether genetically determined as components of a unique syndromic condition.

**Figure 4:**
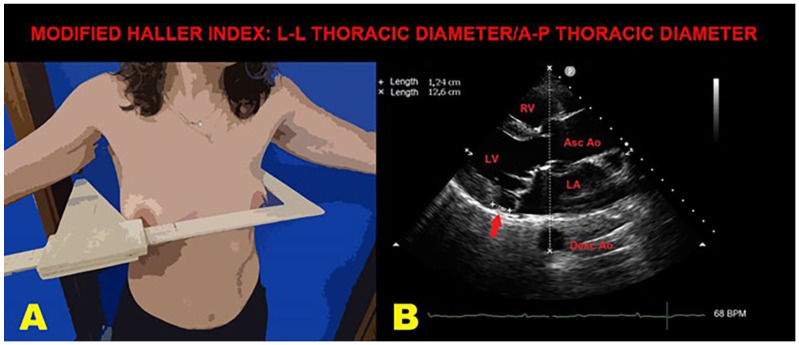
An example of MHI assessment in an MVP individual with MAD. (**A**): The L-L thoracic diameter, measured by using a rigid ruler in centimeters coupled to a level, placed at the distal third of the sternum, at the point of maximum depression of the sternum. (**B**): The A-P thoracic diameter (white dotted line), obtained from the echocardiographic parasternal long-axis view by measuring the distance between the true apex of the sector and the posterior wall of the descending thoracic aorta, visualized behind the left atrium. MAD is indicated by the red arrow. Ao, aorta; A-P, antero-posterior; Asc, ascending; Desc, descending; LA; left atrium; L-L, latero-lateral; LV, left ventricle; MAD, mitral annular disjunction; MHI, modified Haller index; MVP, mitral valve prolapse; RV, right ventricle.

Figure 4 illustrates an example of MHI assessment in an MVP individual with MAD and a narrow A-P thoracic diameter.

### 4.4. Clinical Implications

Individuals with a concave-shaped chest wall, symptoms of palpitations and T-wave inversion in inferolateral leads on ECG, especially if young females with a low cardiovascular disease burden, should always be suspected of having MVP and/or MAD.

Considering the increased risk of complex VAs associated with MAD presence and extent, all MVP patients should be investigated for MAD detection on TTE, and for symptoms suggestive of arrhythmias (especially palpitations and syncope). MVP individuals with MAD should undergo regular 24 h ECG Holter monitoring at diagnosis and during follow-up, with detailed evaluation of arrhythmias [66]. In selected cases, particularly in MVP patients with abnormal electrocardiographic or Holter findings and with no clear evidence of MAD on TTE, a comprehensive multimodality imaging approach involving TEE and/or CMR would add value to individual diagnostic tests. Among the imaging modalities, CMR may better define MAD of minor degree and myocardial fibrosis, allowing clinicians to identify MVP patients with a potential arrhythmogenic substrate [67].

The impairment of myocardial strain parameters, more enhanced at the level of basal myocardial segments, detected by strain echocardiographic imaging in MVP patients with MAD, might not necessarily be an expression of intrinsic myocardial dysfunction. The individual’s chest wall conformation, particularly a narrow A-P thoracic diameter due to sternal depression, might exert a strong influence on cardiac kinetics and function, leading to dis-synchrony as a major determinant of a variable angle of incidence of the ultrasound beam during the cardiac cycle, inducing reduced strain value calculations by the software [68]. It is noteworthy that various degrees of anterior chest wall deformities, ranging from mild concave-shaped chest wall conformation to severe forms of pectus excavatum, may be observed in clinical practice more commonly than expected. An increased prevalence of MVP, nonspecific ST-T-wave abnormalities and VAs, a low probability of CAD and a good outcome over a mid-term follow-up have been demonstrated in individuals with various forms of anterior chest wall deformity [69,70].

The absence of an increased mortality rate over a significant time frame documented by studies conducted on large cohorts of MVP patients [25,28] would suggest that clinicians should avoid uncontrolled therapeutic interventions, such as the use of ICD devices for primary preventive use [29].

MV surgery remains the gold-standard of care for patients with severe MVP and MAD. The presence of MAD does not impact the surgical outcome [29,32], and the surgical correction of MAD appears to be beneficial for reducing the occurrence of VAs after surgery [71].

### 4.5. Limitations of Included Studies

The main limitations of the included studies were the following: (1) the monocentric nature for 82.6% of them; (2) the retrospective design for 87% of them; (3) the use of unadjusted data for 69.6% of them; (4) the lack of follow-up information for 47.8% of them; (5) substantial heterogeneity between the included studies with regard to the MVP cohorts included, affected by various MR degrees and investigated by different imaging modalities. In this regard, it is important to consider that CMR is limited by its time-consuming nature, high cost and reduced availability, TEE is limited by its semi-invasive nature, and, finally, TTE may be affected by its dependence on acoustic windows and low spatial resolution [16]. Finally, only very limited information is available on MAD assessment in MVP individuals by three-dimensional (3D)-TTE or 3D-TEE [30].

## 5. Conclusions

The overall pooled prevalence of MAD in MVP individuals is 40%, with a wide range of variability, depending on the specific imaging method used for assessing MAD presence and on a nonunivocal MAD definition, with a possible overestimation due to Pseudo-MAD rather than True-MAD measurement.

MAD is more commonly associated with female sex, young age, a narrow A-P thoracic diameter, symptoms of palpitations and syncope, T-wave inversion in inferolateral leads and frequent and/or complex VAs, myxomatous leaflets, bileaflet prolapse, larger MVA, non-severe MR and good surgical outcomes.

A multimodality imaging approach associated with noninvasive chest shape assessment might improve MAD detection in MVP individuals.

It appears that careful serial monitoring for arrhythmias should be mandatory for MVP patients with MAD.

Future clinical studies are needed for elucidating the arrhythmic risk associated with Pseudo-MAD and True-MAD phenotypes in MVP individuals.

## Figures and Tables

**Table 1:** Summary and main findings of included studies [7,15,16,24,25,26,27,28,29,30,31,32,33,34,35,36,37,38,39,40,41,42,43]. 3D, three-dimensional; AF, atrial fibrillation; ALS, apical longitudinal strain; A-P, antero-posterior; BD, Barlow’s disease; BLS, basal longitudinal strain; CAD, coronary artery disease; CCT, cardiac computed tomography; CMR, cardiac magnetic resonance; ECV, extracellular volume; eLVH, eccentric left ventricular hypertrophy; FU, follow-up; HF, heart failure; GLS, global longitudinal strain; LASr, left atrial reservoir strain; LAVi, left atrial volume index; LGE, late gadolinium enhancement; LV, left ventricular; LVEF, left ventricular ejection fraction; MACE, major adverse cardiovascular event; MAD, mitral annular disjunction; MLS, mid-longitudinal strain; MR, mitral regurgitation; MV, mitral valve; MVA, mitral valve annulus; MVP, mitral valve prolapse; NR, not reported; NSVT, non-sustained ventricular tachycardia; PMs, papillary muscles; sPAP, systolic pulmonary artery pressure; STE, speckle tracking echocardiography; TAPSE, tricuspid annular plane systolic excursion; TEE, transesophageal echocardiography; TTE, transthoracic echocardiography; VAs, ventricular arrhythmias. The symbol ↑ indicates significantly higher prevalence or magnitude of the single parameter in MAD+ patients vs. MAD− patients; the symbol ↓ indicates significantly lower prevalence or magnitude of the single parameter in MAD+ patients vs. MAD− patients; the symbol ↔ indicates similar (and not statistically different) prevalence or magnitude of the single parameter in MAD+ patients vs. MAD− patients.

Study Name and Country	Number of Patients	Mean Age (yrs)	Females (%)	Imaging Method	Study Design (FU)	MAD Prevalence (%)	Main Findings in MAD+ Patients vs. MAD− Patients
Eriksson, M.J. et al. (2005) [24], Canada	99	52	34	TEE	Retrospective (5.9 yrs)	69.7	linear correlation between magnitude of MAD and number of segments with prolapse/flail; good surgical outcome
Carmo, P. et al. (2010) [15], Portugal	38	57	47.4	TTE	Retrospective	55	↑ females, chest pain paradoxical systolic MVA expansion ↑ NSVT risk when MAD > 8.5 mm
Mantegazza, V. et al. (2019) [25], Italy	979	59	NR	TTE	Retrospective	16.2	younger age, ↑ MVA size, bileaflet MVP and BD ↓ flail; ↔ LV volumes, LVEF, left atrial size ↔ MV repair feasibility
Essayagh, B. et al. (2019) [26], Monaco	89	64	71	CMR	Retrospective	35	younger age; ↑ MVA diameters, myxomatous MVP, bileaflet MVP, non-severe MR ↑ VAs and PM fibrosis ↔ left cardiac chamber size
Putnam, A.J. et al. (2020) [27], USA	90	63	26	CCT	Retrospective	20	↑ females, posterior leaflet length P2 scallop involvement in 89% of cases ↓ end-diastolic MVA area
Mantegazza, V. et al. (2021) [16], Italy	131	57	26.7	TTE, TEE, CMR	Retrospective	17.3, 25.5, 42.0	↑ myxomatous MVP, MAD prevalence in 2C view; ↓ MAD detection and overestimation by TTE; strong agreement between TEE and CMR
Essayagh, B. et al. (2021) [28], USA	595	61	47	TTE	Retrospective (10.3 yrs)	31	younger age; ↓ CAD, HF, hypertension, AF ↑ bileaflet MVP, LV size, palpitations and syncope; ↑ VAs; ↔ overall survival
Bennett, S. et al. (2022) [29], UK	185	66.7	18.3	TTE	Retrospective (4.1 yrs)	32.4	↑ myxomatous MVP, MR degree, inferolateral LV wall involvement; ↓ MAD post-MV surgery ↔ MACE over follow-up
Biondi, R. et al. (2022) [30], France	85	59	45	3D-TEE	Retrospective	38.8	↑ P2 scallop involvement MAD phenotypes: bimodal (63.6%) or flat (36.4%); good surgical outcome
Figliozzi, S. et al. (2023) [7], Europe	474	47	51.5	CMR	Retrospective (3.2 yrs)	68	younger age, ↑ chest pain; ≥10,000 VAs/day LGE prevalence and extent associated with adverse outcome
Sonaglioni, A. et al. (2023) [31], Italy	93	54.2	50.5	TTE, STE	Prospective	34.4	↓ A-P thoracic diameter; ↑ myxomatous MVP ↓ cardiac chambers cavity sizes ↔ LVEF; ↓ LV-GLS and LV-GCS
Gray, R. et al. (2023) [32], Australia	111	66.3	29.7	TTE	Retrospective (3.9 yrs)	28.8	younger age, ↑ females, ↓ CAD ↑ myxomatous MVP, bileaflet MVP, MVA diameters; ↑ inferior T-wave inversion, severe MR; ↓ MAD and ↑ VAs post-MV surgery
Hussain, N. et al. (2023) [33], USA	100	58	55	CMR	Retrospective (2.1 yrs)	52	↑ bileaflet MVP, paradoxical systolic MVA expansion; ↑ abnormal LGE ↑ VT risk when MAD ≥ 4 mm + abnormal LGE
Vaksmann, G. et al. (2023) [34], France	49	12.8	67	TTE	Prospective	51	↑ T-wave inversion in inferolateral leads ↑ VAs in presence of Pickelhaube sign, myxomatous MVP and dilated left ventricle
Shechter, A. et al. (2023) [35], USA	271	82	39.1	TTE, STE	Retrospective (1 year)	22.9	↓ LV chamber cavity sizes; ↔ LVEF, LV-GLS ↑ bileaflet MVP, posterior leaflet, MVA ↑ procedural time and mortality risk
Perazzolo Marra, M. et al. (2024) [36], Italy	108	48	61	CMR	Retrospective (3.7 yrs)	57	↑ LGE in LV-basal inferolateral wall and PMs ↑ VAs in presence of greater MAD distance, curling and LGE
Özyıldırım, S. et al. (2024) [37], Turkey	103	36	50	TTE, STE	Prospective	33	↑ symptoms, T-negativity in inferior leads ↑ MR degree, MVA diameters, Pickelhaube sign and LGE; ↓ LV-GLS, LV-BLS, LV-MLS; ↔ LV-ALS; ↓ LASr
Blondeel, M. et al. (2024) [38], Belgium	12	51.3	75	CMR	Retrospective (4.7 yrs)	67	↑ VAs in presence of new LGE (fibrosis) in mid- to basal inferolateral wall and both PMs on repeat CMR after median of 4.7 yrs
Meucci, M.C. et al. (2024) [39], the Netherlands	231	48	55	TTE, STE	Retrospective (3.1 yrs)	39	↑ eLVH, LAVi, MVA, TAPSE; ↓ LASr ↔ LVEF, LV-GLS, E/A, E/e’, sPAP ↑ MR progression
Cesmat, A.P. et al. (2024) [40], USA	632	62	52.7	TTE	Retrospective (3.3 yrs)	14.9	↑ females, T-wave inversion in inferolateral leads; ↑ myxomatous bileaflet MVP; ↓ hypertension; ↑ complex VAs and all-cause mortality risk
Figliozzi, S. et al. (2024) [41], Italy	29	55	42	CMR	Retrospective (1 year)	90	younger age, ↓ CAD; ↓ LV dimensions, ↑ LVEF; ≥10,000 VAs/day; MAD positively correlated with MVP extent and systolic MVA
Palmisano, A. et al. (2024) [42], Italy	2611	53	34	CMR	Retrospective (5 yrs)	5.4	↑ MAD length, ECV, systolic curling in MAD patients with bileaflet MVP; ↑ arrhythmias in case of MAD ≥ 5 mm and bileaflet MVP
Fiore, G. et al. (2025) [43], Italy	603	52	50	TTE, STE, CMR	Retrospective	7	younger age, ↑ females ↑ LV end-diastolic volumes; ↑ degree of MR ↑ periannular fibrosis (True-MAD) ↑ inferolateral fibrosis (Pseudo-MAD)

**Table 2:** Clinical and instrumental characteristics of MVP patients analyzed by included studies. Data are expressed as median and IQR. ACEi, angiotensin-converting enzyme inhibitor; AF, atrial fibrillation; ALS, apical longitudinal strain; A-P, antero-posterior; ARBs, angiotensin II receptor blockers; BLS, basal longitudinal strain; BMI, body mass index; BSA, body surface area; CAD, coronary artery disease; CCT, cardiac computed tomography; CHF, congestive heart failure; CMR, cardiac magnetic resonance; EDD, end-diastolic diameter; ESD, end-systolic diameter; EDV, end-diastolic volume; ESV, end-systolic volume; GLS, global longitudinal strain; IQR, interquartile range; IVS, interventricular thickness; LASr, left atrial reservoir strain; LAVi, left atrial volume index; LGE, late gadolinium enhancement; LV, left ventricular; LVEF, left ventricular ejection fraction; LVMi, left ventricular mass index; MAD, mitral annular disjunction; MHI, modified Haller index; MLS, mid-longitudinal strain; MR, mitral regurgitation; MVA, mitral valve annulus; MVP, mitral valve prolapse; NSVT, non-sustained ventricular tachycardia; PW, posterior wall; RV, right ventricular; RVEF, right ventricular ejection fraction; sPAP, systolic pulmonary artery pressure; TAPSE, tricuspid annular plane systolic excursion; TEE, transesophageal echocardiography; TTE, transthoracic echocardiography.

	Average Value (IQR)	Number of Studies for Parameters Assessed (%)
**Demographics**		
Age (yrs)	55 (12.8–82)	23 (100)
Female sex (%)	46.7 (18.3–75)	22 (95.6)
**Anthropometrics**		
BSA (m^2^)	1,9 (1.8–1.97)	5 (21.7)
BMI (Kg/m^2^)	25.1 (23.5–29)	5 (21.7)
MHI	2.3 (2.0–2.6)	1 (4.3)
**Cardiovascular risk factors and cardiovascular disease burden**		
Hypertension (%)	31.1 (12–78.2)	11 (47.8)
Smokers (%)	18.8 (3.4–41.7)	8 (34.8)
Dyslipidemia (%)	22.8 (8–43.2)	10 (43.5)
Type 2 diabetes (%)	7 (0.5–18.6)	11 (47.8)
CAD history (%)	10.4 (1–27.3)	11 (47.8)
CHF history (%)	15.9 (3.3–35.1)	6 (26.1)
**Symptoms (%)**		
Dyspnea (%)	22.4 (5.7–42)	6 (26.1)
Palpitations (%)	35.9 (8–58.8)	9 (39.1)
Fatigue (%)	16.4 (11.8–21)	2 (8.7)
Chest pain (%)	16 (7–29)	7 (30.4)
Syncope (%)	10.8 (1–23)	9 (39.1)
**ECG/24 h Holter monitoring findings**		
T-wave inversion in inferolateral leads (%)	29.2 (20–46.2)	6 (26.1)
Atrial arrhythmias and/or AF (%)	20 (1.1–55)	15 (65.2)
Ventricular arrhythmias (%)	33.4 (6.3–61.4)	11 (47.8)
NSVT (%)	11.8 (1.8–33)	7 (30.4)
**Conventional echoDoppler variables**		
IVS thickness (mm)	9.7 (9–10)	3 (13)
PW thickness (mm)	9.4 (9–9.8)	2 (8.7)
LV-EDD (mm)	51.5 (41.3–60)	9 (39.1)
LV-ESD (mm)	33.2 (31–39)	6 (26.1)
LVMi (g/m^2^)	83.5 (54–119.1)	7 (30.4)
LV-EDV (mL/m^2^)	79.3 (43.1–107)	8 (34.8)
LV-ESV (mL/m^2^)	30 (15.8–39)	8 (34.8)
LVEF (%)	60.9 (48.5–66)	18 (78.3)
LAVi (mL/m^2^)	48.8 (31–82)	11 (47.8)
E/A ratio	1.0 (0.83–1.2)	2 (8.7)
E/e’ ratio	8.5 (7–10)	2 (8.7)
RVIT (mm)	34 (29.1–39)	2 (8.7)
TAPSE (mm)	22.2 (18–25)	4 (17.4)
sPAP (mmHg)	31.9 (25–43)	6 (26.1)
**Mitral valve parameters**		
Bileaflet MVP (%)	46 (2.4–79)	16 (69.6)
Posterior leaflet prolapse only (%)	43.9 (21–85)	8 (34.8)
MVA end-systolic A-P diameter (mm)	35.9 (29.6–44.5)	9 (39.1)
Myxomatous morphology (%)	60.8 (36.9–75)	4 (17.4)
Mild-to-moderate MR (%)	40.7 (12.5–100)	15 (65.2)
Severe MR (%)	55 (34–100)	8 (34.8)
Accompanying flail (%)	27.9 (6–48)	6 (26.1)
MAD overall prevalence (%)	39.6 (5.4–90)	23 (100)
MAD prevalence in TTE studies (%)	31.3 (14.9–55)	12 (52.2)
MAD prevalence in TEE studies (%)	44.7 (25.5–69.7)	3 (13)
MAD prevalence in CMR studies (%)	47 (5.4–90)	9 (39.1)
MAD prevalence in CCT studies (%)	20	1 (4.3)
MAD extent (mm)	7.1 (4.2–10)	21 (91.3)
Females with MAD (%)	60 (52–66)	13 (56.5)
Pickelhaube sign (%)	24.7 (6.5–37)	5 (21.7)
**Myocardial strain parameters (%)**		
LV-GLS (%)	19.3 (15.9–23.1)	6 (26.1)
LV-BLS (%)	14.6 (11–19.6)	3 (13)
LV-MLS (%)	19.3 (16.4–22.2)	2 (8.7)
LV-ALS (%)	24.8 (22.1–27.5)	2 (8.7)
LASr (%)	27.9 (24.5–31.2)	2 (8.7)
**CMR findings**		
LV-EDV (mL/m^2^)	86.2 (77.1–95)	3 (13)
LV-ESV (mL/m^2^)	34.8 (31.5–38)	3 (13)
LVEF (%)	59.3 (59–60)	3 (13)
RV-EDV (mL/m^2^)	79 (72.6–82)	4 (17.4)
RV-ESV (mL/m^2^)	31.7 (29–33)	4 (17.4)
RVEF (%)	60 (57–62)	4 (17.4)
Abnormal LGE (%)	35.9 (6.1–68)	10 (43.5)
**Current medical treatment**		
Beta blockers (%)	32.6 (14–58.7)	8 (34.8)
ACEi/ARBs (%)	28.1 (14.9–62)	13 (56.5)
Antiarrhythmics (%)	15.4 (13–17.8)	2 (8.7)

## Data Availability

Data extracted from the included studies will be publicly available on Zenodo (https://zenodo.org) (accessed on 31 October 2024), pending acceptance by the journal.
